# Antibacterial Efficacy Comparison of Electrolytic and Reductive Silver Nanoparticles Against *Propionibacterium acnes*

**DOI:** 10.3390/antibiotics14010086

**Published:** 2025-01-14

**Authors:** Suparno Suparno, Rita Prasetyowati, Khafidh Nur Aziz, Anggarwati Rahma, Eka Sentia Ayu Lestari, Siti Chaerani Nabiilah, Deby Grace

**Affiliations:** Department of Physics Education, Faculty of Mathematics and Science, Universitas Negeri Yogyakarta, 1st Colombo St., Karangmalang, Sleman, Yogyakarta 55281, Indonesia; rita.p@uny.ac.id (R.P.); khafidh.na@uny.ac.id (K.N.A.); rahmaaw99@gmail.com (A.R.); ekasentiaayulistari@gmail.com (E.S.A.L.); schanabiilah@gmail.com (S.C.N.); debygracesl@gmail.com (D.G.)

**Keywords:** reduction, toxic free electrolysis, antibiotics, bacterial resistance, Kirby–Bauer method

## Abstract

**Background:** The aim of this study was to develop an electrolysis system to produce silver nanoparticles free from toxic gases, as the most common reduction and electrolysis techniques produce nitrogen dioxide (NO_2_) as a byproduct, which is harmful to human health. The new electrolysis system used two identical silver plate electrodes, replacing silver and carbon rods, and used water as the electrolyte instead of silver nitrate (AgNO_3_) solution since AgNO_3_ is the source of NO_2_. **Methods:** The electrolytic silver nanoparticles (ESNs) produced by the new system were characterized and compared with reductive silver nanoparticles (RSNs). Using UV–Visible spectrophotometry, absorption peaks were found at 425 nm (ESN) and 437 nm (RSN). Using dynamic light scattering, the particle diameters were measured at 40.3 nm and 39.9 nm for ESNs at concentrations of 10 ppm and 30 ppm, respectively, and 74.0 nm and 74.6 nm for RSNs at concentrations of 10 ppm and 30 ppm, respectively. Antibacterial activity against *Propionibacterium acnes* (*P. acnes*) was assessed using the Kirby–Bauer method. **Results:** It was found that the efficacy of ESNs and RSNs was relatively lower than that of 5% chloramphenicol because it was measured in different concentration units (ESNs and RSNs in ppm and chloramphenicol in %). Using the calibration curve, the efficacy of 5% chloramphenicol was comparable to that of 0.005% ESN. It was also found that *P. acnes* developed a strong resistance to chloramphenicol and showed no resistance to ESNs. **Conclusions:** This finding underlines the tremendous potential of ESNs as a future antibiotic raw material.

## 1. Introduction

Most bacteria develop drug resistance due to drug abuse, drug overuse, and long-term exposure to the same drug [1,2]. Using the wrong type of antibiotic will not cure the disease. It can even cause resistance to antibiotics. Excessive use of drugs can kill some bacteria but can lead to resistance. Prolonged use of drugs can cause bacteria to develop resistance [3]. The development of bacterial resistance to many antibacterial agents is rapid [3,4]. Meanwhile, the discovery of new and more effective drugs is very slow [5].

*Propionibacterium acnes* (*P. acnes*) is a Gram-positive bacterium commonly found on the skin, in the oral cavity, and in the gastrointestinal tract [6]. This bacterium is aerobic, aerotolerant, and commensal, and under certain conditions, it turns into a pathogen [7]. *P. acnes* and Staphylococcus aureus are the main pathogens involved in acne, and they are hated by millions of young people who do not want their faces to look ugly due to *P. acnes* infection. *P. acnes* has been observed to develop resistance to many antibiotics over the past 40 years. In 1983, Denys and colleagues showed that *P. acnes* only showed resistance to metronidazole among 22 different antibiotics used in their study, including tetracycline, erythromycin, and clindamycin [8]. However, recent studies have shown resistance of *P. acnes* to tetracycline, erythromycin, and clindamycin [9,10]. Many other reports have shown the development of *P. acnes* resistance to various antibiotics, such as azithromycin, clarithromycin, and trimethoprim-sulfamethoxazole [11,12]. *P. acnes* was chosen for this study because of the continued development of resistance to various types of antibiotics and the high market demand for antibiotics to combat *P. acnes* resistance.

Research in the development of antibiotic raw materials has been going on for decades. Medicinal plants and metal nanoparticles have been explored as antibiotic raw materials [13,14,15,16]. Many studies on silver nanoparticles have been carried out over the last 2–3 decades for various purposes, such as chemical sensors [17,18,19], conductive inks [20] nano-fertilizers and nano-pesticides [21], textiles [22,23], drug delivery [24,25], and antibacterial agents [15,26]. Plant-based antibiotics are relatively easy to prepare but take a long time to reproduce, and it is difficult to maintain the sustainability of the raw materials. On the other hand, metal nanoparticles are relatively difficult to prepare and easy to reproduce, and the raw materials are abundant. Silver nanoparticles were chosen for this study because they have been widely studied, so it is easier to compare our research with published papers, and they are low cost and show high efficacy [16,27]. However, we must contend with the cytotoxicity of silver nanoparticles, as pointed out by Bruna [15].

**Figure 1:**
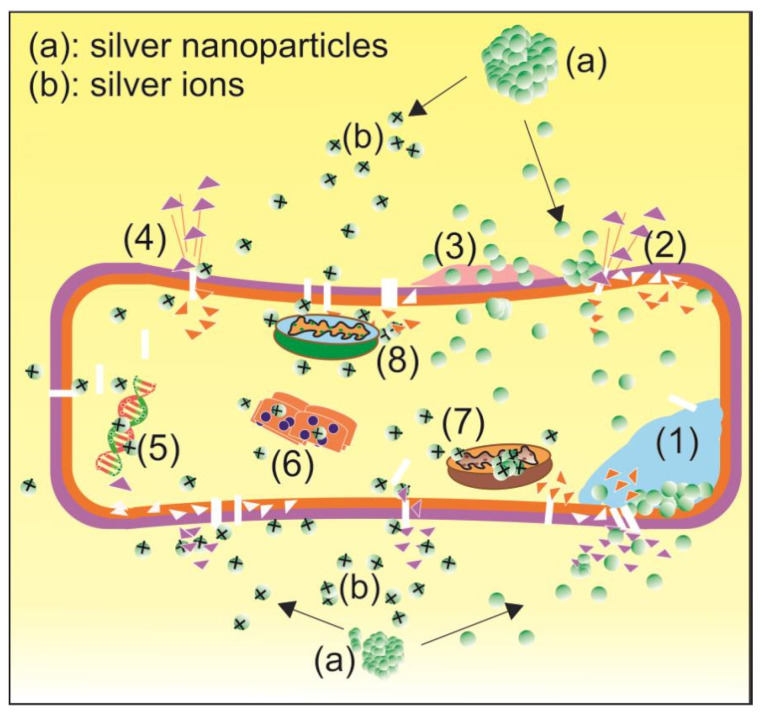
Possible mechanisms of silver nanoparticles in killing and inhibiting bacteria: (1) cytoplasmic membrane denaturation due to silver nanoparticle accumulation, (2) membrane structure disruption due to nanoparticle penetration, (3) inhibition of *P. acnes* biofilm production, (4) silver ions released by silver nanoparticles damage the cell wall and penetrate cytoplasm, (5) silver ions interfere with the production of adenosine triphosphate leading to low energy production, (6) silver ions cause denaturation of ribosomes and the cytoplasm and disrupt protein synthesis, (7) respiratory enzyme inactivation by silver ions, leading to bacteria suffocation, and (8) increase in reactive oxygen species (ROS) production caused by silver ions penetration causes oxidative stress to most of the internal organs of the bacteria. As for color: The green balls represent silver nanoparticles, the green balls with a plus symbols represent Ag^+^ ions, the pink represents biofilm, the blue represents denaturation of the cytoplasmic membrane, the yellow inside the bacterial cell wall is cytoplasm, the orange triangles represent membrane fragments, and the pink triangles are cell wall fragments.

The relatively high efficacy of silver nanoparticles is most likely due to their bactericidal properties. Silver nanoparticles not only inhibit the growth of bacteria but also kill them. Although the exact mechanism is still unknown, there are eight proposed mechanisms by which silver nanoparticles inhibit and kill bacteria, as depicted in Figure 1. There are two types of silver nanoparticle soldiers involved in fighting bacteria, namely “regular soldier” silver nanoparticles (a) and “special force” silver ions (b). They fight bacteria in eight different ways simultaneously, making it difficult for bacteria to develop resistance [15,28,29].

There are two main techniques for producing silver nanoparticles, namely reduction and electrolysis techniques [20,30]. The reduction technique is carried out by mixing silver nitrate solution and trisodium citrate solution. Both solutions are mixed and heated in water using a magnetic stirrer. The reduction technique can produce reductive silver nanoparticles (RSN) quickly [31]. However, there are two problems that are not realized by most people. First, this technique produces byproducts in the form of citric acid and sodium hydroxide [32]. Citric acid contains three carboxyl functional groups known as auxochromes per molecule that shift the absorption peak to the red. These auxochromes absorb some of the transitional energy so that the absorption peak wavelength shifts to red, i.e., to lower energy. Second, this technique produces a byproduct of the toxic gas nitrogen dioxide, NO_2_ [33,34]. NO_2_ is a dangerous gas produced from the decomposition of HNO_3_ when silver nitrate is used as a precursor. On the other hand, the electrolysis technique using silver nitrate as an electrolyte produces a silver solution that is free from contaminants (such as citric acid) but still produces the same toxic gas, NO_2_. This inspired us to create a modified electrolysis technique to produce silver nanoparticles free from contaminants and toxic gases and observe their antibacterial properties.

The formation of silver nanoparticles using electrolytic and reductive techniques is highly dependent on the aggregation of silver atoms in solution [35]. The higher the concentration of silver atoms, the larger the size of silver nanoparticles. It is estimated that each silver nanoparticle contains 20 to 15,000 silver atoms [36]. The short-range van der Waals attraction is the most likely cause of aggregation when atoms collide with each other due to Brownian motion [37]. Therefore, we believe that the physical characteristics of electrolytic silver nanoparticles (ESN) and RSN will be the same unless the environment around the silver nanoparticles changes them.

The antibacterial activity of ESNs and RSNs should be similar, largely due to the silver nanoparticles themselves, the release of Ag^+^ ions, and the production of reactive oxygen species (ROS), which are all summarized in Figure 1. However, it will be more difficult for RSNs to release Ag^+^ ions due to the presence of a layer of citric acid byproduct on the surface of RSNs, which can reduce its efficacy. Positive ions are attracted to the bacterial cell wall because its surface is negatively charged due to the presence of polyanionic teichoic acids (bound to peptidoglycan), which make up 50% of the mass of the bacterial cell wall [38,39]. The attachment of Ag^+^ to the cell wall can cause the wall to increase its permeability and leak its contents, which leads to bacterial death. The leakage of protein-rich bacterial contents can be measured using the Bradford colorimetric protein assay, which is based on the absorbance of Coomassie G-250 brilliant blue dye [40,41]. The dye binds to the leaked proteins, causing the absorption peak to shift from 465 nm to 595 nm [42]. The absorbance observed by visible spectroscopy at 595 nm is proportional to the concentration of leaked proteins. The leakage from bacterial cell walls allows Ag^+^ and ESNs to penetrate, attach to, and disrupt the function of many internal organs, causing inhibition of bacterial growth or death. On the other hand, the transport of Ag^+^ ion causes intracellular ROS and free radicals to increase [43], which eventually damages lipids, proteins, and DNA. The increase in ROS consisting of many free radicals can be observed using electron spin resonance (ESR). Each free radical shows a specific ESR spectrum, and the signal strength of the ESR spectrum is proportional to the concentration of free radicals.

For now, we are more interested in exploring the biological characteristics of ESNs and RSNs, especially their antibiotic properties. Therefore, observation of the antibacterial activity of ESNs and RSNs was carried out and compared with that of chloramphenicol as a positive control. The focus of the observation was on the efficacy (as indicated by the diameter of the clear zone) of ESNs compared to RSNs against P. acnes [44,45]. However, its power to prevent the development of bacterial resistance to ESNs and RSNs was also observed [46].

The presence of silver atoms in the solution was observed using UV–Visible spectroscopy [47]. The size of the silver atoms was determined using dynamic light scattering [48]. The efficacy and strength to prevent the development of resistance were observed using the Kirby–Bauer method [49]. The efficacy of ESNs and RSNs at 10 ppm and 30 ppm was compared, a *t*-test analysis was performed, and the results are discussed thoroughly in this paper. The results were also compared with those using 5% chloramphenicol as a positive control [50].

## 2. Results

### 2.1. Comparison of Solution Colors

**Figure 2:**
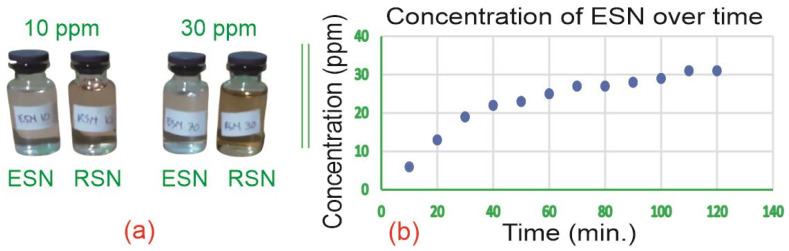
(**a**) ESN and RSN color and (**b**) ESN concentration over time. The blue dots represent the concentration of ESN (in ppm) at certain times (in minute) which were written in green color in the horizontal.

Figure 2a shows photos of four samples of ESNs (10 ppm and 30 ppm) and RSNs (10 ppm and 30 ppm) for color comparison. Figure 2b shows the increase in ESN concentration (ppm) over time (minutes). The color differences will be discussed later.

### 2.2. Silver Content in Solution

**Figure 3:**
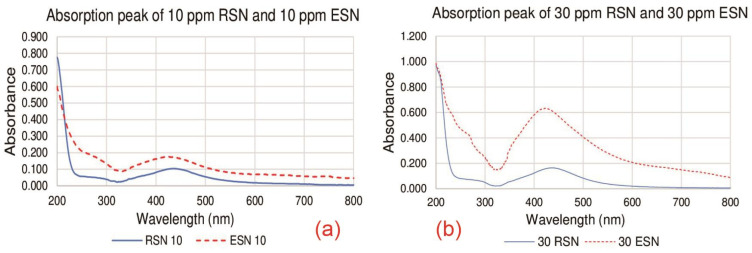
Absorption peaks: (**a**) 10 ppm and (**b**) 30 ppm of ESNs and RSNs.

Figure 3 depicts the results of the UV–Visible spectrometer measurements on the absorption peak wavelengths of ESNs and RSNs at 10 ppm (a) and 30 ppm (b). The possible causes of the different peaks will be discussed.

### 2.3. Particle Size Comparison

Table 1 shows data on average particle sizes of ESNs and RSNs and the polydispersity index (PDI) of the ESN and RSN solutions.

### 2.4. Efficacy and Power to Prevent Resistance

**Figure 4:**
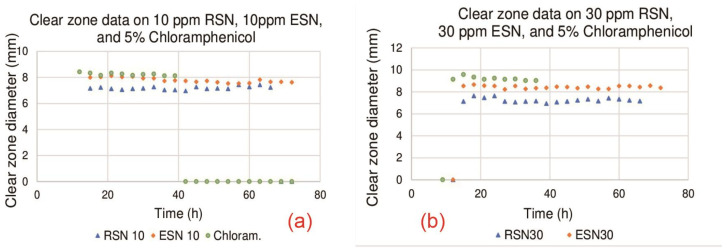
Clear zone diameter of (**a**) 10 ppm and (**b**) 30 ppm of all antibiotics.

Figure 4a shows the results of clear zone diameter measurements of ESN (10 ppm), RSN (10 ppm), and chloramphenicol (5%) in the stationary stage. Figure 4b shows the results of the clear zone diameter measurements of ESNs (30 ppm), RSNs (30 ppm), and chloramphenicol (5%) in the stationary stage. Table 2 shows the two-tailed *p*-values from the statistical analysis.

## 3. Discussion

### 3.1. Comparison of Solution Colours

Figure 2a shows the color difference of the ESN and ESN solutions. The leftmost sample is 10 ppm ESN, and the sample next to it is 10 ppm RSN. It can be clearly seen that the 10 ppm ESN solution is much clearer than the 10 ppm RSN solution. The RSN (10 ppm) solution is slightly yellowish. The same color difference also occurred between the 30 ppm ESN and 30 ppm RSN (rightmost) solutions. Here, 30 ppm RSN is much more reddish yellow than all the other samples. This is most likely due to the presence of NO_2_ gas, which is yellowish-brown and slightly soluble in water (see Equation (3)) [51]. As for the ESN solution, the color of the solution was relatively unchanged because there were no remaining byproducts, including NO_2_, in the solution. The clear ESN solution, like water, indicates the purity of ESN, which is free from byproducts and contaminants.

### 3.2. Silver Content in Solution

UV–Visible spectrophotometry showed that the absorption peak wavelengths of the 10 ppm ESN and RSN solutions were 425 nm and 437 nm, respectively, as shown in Figure 3a. The same peaks were shown for the 30 ppm ESN and RSN solutions in Figure 3b. The peak wavelengths between 400 and 450 nm indicate the presence of silver [52]. Each graph shows only one peak, which means that only silver atoms contribute to the light absorption in the ESN and RSN solutions. The data in Figure 3a,b show three interesting phenomena to discuss. First, both concentrations of ESNs showed the same peak at 425 nm [53], while both concentrations of RSNs showed the same peak at 437 nm [54]. This means that the dilution process did not change the contents of ESNs and RSNs.

Second, there is a difference in the peak wavelength of absorption between ESNs and RSNs. This means that the peak wavelength of RSNs is shifted to the right (redshift of 12 nm). This is most likely related to the presence of bathochromic carboxyl in the RSN solution [55], as there are three carboxyl functional groups and one hydroxyl functional group in each molecule of citric acid byproduct, and carboxyl and hydroxyl are known as bathochromic auxochromes that shift the peak to the red, i.e., to a larger wavelength. Finally, the absorbance of the RSN solution is lower than that of the ESN solution at both concentrations. This low absorbance is due to the hypochromic effect of the hydroxyl functional group [56]. Some chromophores, such as hydroxyl, cause hypochromic effects by absorbing more light intensity. The absorption of light intensity by hydroxyl causes the absorbance to decrease. In addition, low pH is known to cause hypochromic effects by reducing absorbance. The RSN solution contains citric acid, so its pH is lower than that of the ESN solution. Kiani et al. showed a hypochromic effect on pH can be up to 34% [57].

### 3.3. Particle Size Comparison

The data presented in Table 1 show that the diameters of both types of silver nanoparticles remain relatively stable across a range of concentrations, indicating that adding a small amount of water to dilute the silver nanoparticle solution does not change the particle size. Table 1 also shows that the RSN diameter is significantly larger than the ESN diameter. This may be due to the presence of reductants in the solution that help accelerate the aggregation of silver nanoparticles. The smaller size of the ESNs may be attributed to the slow electrolysis process, which leads to the slow formation of silver atoms, silver atom aggregation, and ESN production. As the electrolysis progresses, the formation of bubbles on the cathode increases, and the cathode appears darker. The bubbles and dark layers on the cathode surface inhibit the formation of silver atoms [58]. Figure 2b shows that 22 ppm of ESN was produced during the first 40 min, but only 9 ppm was produced during the next 80 min. Meanwhile, the simultaneous redox reaction between AgNO_3_ and Na_3_C_6_H_5_O_7_ resulted in the rapid production and aggregation of RSNs. Therefore, the RSN sizes (10 ppm and 30 ppm) were significantly larger than the ESN sizes (10 ppm and 30 ppm).

The PDI of the RSNs (0.2848 and 0.2948) was also larger than that of the ESNs (0.0533 and 0.0642). This indicates that the size distribution of RSNs is more heterogeneous than that of ESNs [59]. It is understandable that the ESN solution is highly homogeneous because unlike the RSN solution, no byproducts are left in the ESN solution. The average particle size and PDI data are presented in Supplementary Material Data S1.

### 3.4. Efficacy and Power to Prevent Resistance

To explain the analysis of antibacterial activity data, we introduce three stages of antibacterial action to combat bacteria growing on nutrient-rich agar (NA) plates. The first stage is the initial stage [60]. This stage is relatively short, starting with the placement of the antibacterial-impregnated disc and ending with the onset of the stationary stage. The early stage usually lasts only a few hours. The second stage is the stationary stage. At this stage, the number of dead bacteria is approximately equal to the number of bacteria formed. The diameter of the clear zone is relatively stable, and the diameter of the clear zone indicates the efficacy of the antibacterial agent. If bacteria develop resistance to the antibacterial agent, this stage is relatively short, and the antibacterial agent becomes ineffective. Therefore, the duration of the stationary stage indicates the strength of the antibacterial agent in combatting antibacterial resistance [61]. For an antibacterial agent to be considered strong against resistance, the stationary stage must last longer, thus delaying the final stage. The final stage begins when the diameter of the clear zone continues to decrease due to the development of resistance associated with stronger bacterial growth [62] until the clear zone disappears completely. With an understanding of these three stages, it makes sense to compare the efficacy of different antibacterial agents during this stationary stage.

Figure 4a shows the stationary stage for the 10 ppm RSN and ESN solutions and 5% chloramphenicol. All three antibacterial agents showed antibacterial activity, as indicated by their clear zone diameters. However, the ESN solution showed stronger efficacy than the RSN solution, as evidenced by its consistently larger diameter and longer duration in preventing the spread of *P. acnes*. Statistical analysis showed that 10 ppm ESN produced a significantly larger clear zone diameter than 10 ppm RSN, as indicated by the two-tailed *p*-value of 2.26 × 10^−13^ (see Table 2, row 6 column 5), which was less than 0.05, and thus the null hypothesis (Ho) was rejected. Chloramphenicol showed better efficacy than 10 ppm ESN and 10 ppm RSN, as indicated by its larger clear zone diameter. The *p*-values of the two comparisons between chloramphenicol and 10 ppm ESN (4.81 × 10^−18^) and between chloramphenicol and 10 ppm RSN (2.4 × 10^−9^) were less than 0.05, so Ho was rejected, which means chloramphenicol had significantly greater efficacy than 10 ppm ESN and 10 ppm RSN (see Table 2, row 2, column 5 and row 4, column). The statistical analysis is provided in Supplementary Material Data S2.

Figure 4b shows the stationary stage for the 30 ppm ESN and RSN solutions and 5% chloramphenicol. All three antibacterial agents showed antibacterial activity, indicating the potential of ESNs and RSNs as antibacterial agents. The *t*-test analysis showed that the *p*-values of the comparison between 5% chloramphenicol and 30 ppm ESN and between 5% chloramphenicol and 30 ppm RSN were 3.91 × 10^−8^ and 4.61 × 10^−16^, respectively (see Table 2, row 3, column 5 and row 5, column 5). Both *p*-values were less than 0.05, so Ho was rejected, which means that 5% chloramphenicol had significantly higher efficacy than 30 ppm ESN and RSN. When comparing 30 ppm ESN and RSN, the two-tailed *p*-value of the comparison was 7.91 × 10^−20^, which is lower than 0.05, meaning that 30 ppm ESN produced significantly higher efficacy than 30 ppm RSN.

The *p*-value of statistical analysis of the comparison between the 30 ppm and 10 ppm RSN solutions was 0.254671 (Table 2, row 9, column 5), which is higher than 0.05. Therefore, Ho was accepted, meaning that 30 ppm RSN did not have significantly greater efficacy than 10 ppm RSN. On the contrary, 30 ppm ESN had significantly higher efficacy than 10 ppm ESN, as indicated by the *p*-value of 3.22 × 10^−14^, which is less than 0.05 (see Table 2, row 8, column 5). This means that a higher ESN concentration produces higher efficacy. This trend can be used to estimate the efficacy of ESN at higher concentrations. However, our data are limited to 10 ppm and 30 ppm ESN, so they are not sufficient to draw a reliable calibration curve. Assuming that two data points are sufficient to draw a calibration curve results in a linear regression equation of Y = 0.0314X + 7.4936. Using this equation, an ESN concentration of 54.34 ppm would result in a clear zone diameter equivalent to that of 5% chloramphenicol, which is 9.2 mm. It should be noted that this ESN concentration is very low since 54.34 ppm equals approximately 0.005%, which is much lower than that of 5% chloramphenicol. The superiority of ESNs over chloramphenicol is probably due to the bactericidal property of pure ESNs compared to the bacteriostatic nature of chloramphenicol. ESN kills and inhibits bacteria through many different mechanisms, as depicted in Figure 1. These findings convincingly show the superiority of ESNs as an antibacterial agent over chloramphenicol, which underscores their compelling potential as raw material for future antibacterial agents.

Figure 4a,b also show that the effectiveness of chloramphenicol only lasted 39 h and 36 h, respectively. On the other hand, both figures show that ESNs and RSNs remained effective for 72 h and 66 h, respectively. These results indicate that *P. acnes* exhibits strong resistance to chloramphenicol, making this antibacterial agent ineffective against *P. acnes* after 39 h [63]. This suggests that while chloramphenicol initially has higher efficacy, its effectiveness decreases more rapidly due to antibacterial resistance [64]. Many researchers have shown that *P. acnes* is resistant to many antibiotics [9,10,11,12]. By contrast, *P. acnes* exhibits weaker resistance to RSNs starting at 66 h and does not show resistance to ESNs until the end of observation at 72 h. Both figures indicate that the ESN solution not only maintains its antibacterial activity for a longer period but also shows the power to prevent the development of resistance in *P. acnes*. The ability of the ESN solution to maintain its efficacy without eliciting antibacterial resistance highlights its potential advantages over RSN solution and chloramphenicol.

## 4. Materials and Methods

### 4.1. RSN Production

The precursor 100 mL of 10 mM silver nitrate (${AgNO}_{3}$) was prepared by dispersing 170 mg of ${AgNO}_{3}$ powder with water to a total volume of 100 mL. The mixture was homogenized by stirring on a magnetic stirrer at 50 rpm 25 °C for 5 min. The 100 mL of 10 mM stabilizer was prepared by mixing 258 mg ${Na}_{3}C_{6}H_{5}O_{7}$ with water up to 100 mL. No specific reducing agent was introduced since trisodium citrate is also a reducing agent [63]. The balance reaction is given by:

(1) $$12AgNO_{3}+4{Na}_{3}C_{6}H_{5}O_{7}+6H_{2}O↔12Ag+12NaNO_{3}+4C_{6}H_{8}O_{7}+3O_{2}$$

According to Equation (2), in a single reaction, this technique produces 12 silver atoms, ${12NaNO}_{3}$, ${4C}_{6}H_{8}O_{7}$, and 3 oxygen gas atoms. All of them remain in the solution except oxygen gas. Citric acid, with the chemical formula HOC(${CO}_{2}H$)(${CH}_{2}{CO}_{2}H{)}_{2}$, contains three carboxyl (${CO}_{2}H$) and hydroxyl (OH) functional groups [64]. Citric acid is soluble in water and considered a contaminant in the RSN solution since there is no easy method to separate citric acid from water as a solvent of the RSN solution.

Another major byproduct, ${NaNO}_{3}$, is soluble in water, and it dissociates in water to form ${Na}^{+}$ and ${NO}_{3}^{-}$. This ${NO}_{3}^{-}$ in water reacts quickly with $H^{+}$ ions to form ${HNO}_{3}$, which then decomposes to form ${NO}_{2}$, $H_{2}O$, and $O_{2}$. Here is the complete reaction:

(2) $$\begin{array}{c} 4{NaNO}_{3}↔4{Na}^{+}+4{NO}_{3}^{-}\\ 4H_{2}O↔4H^{+}+4OH^{-}\\ 4{Na}^{+}+{4OH}^{-}↔4NaOH\\ 4H^{+}+{4NO}_{3}^{-}↔4{HNO}_{3}\\ 4{HNO}_{3}↔{4NO}_{2}+{2H}_{2}O+O_{2}\\ {4NaNO}_{3}+{2H}_{2}O↔{4NO}_{2}+4NaOH+O_{2}. \end{array}$$

**Figure 5:**
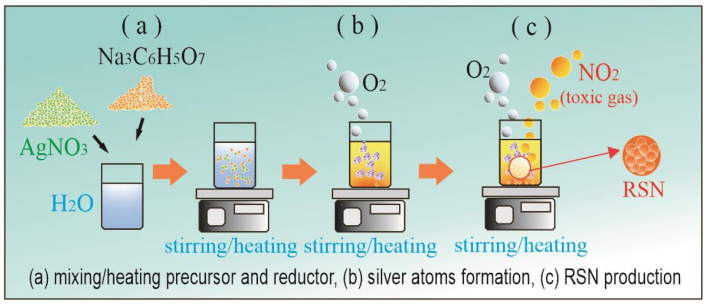
RSN production process: (**a**) mixing/heating precursor and reductor, (**b**) silver atom formation, and (**c**) RSN production. White solvent and many orange dots represent the mixture of silver nitrate and trisodium citrate at the beginning of process. Yellow solvent and many larger dots represent the formation of silver atoms. Yellow solvent and orange bubbles represent nitrogen dioxide formation.

The ${NO}_{2}$ byproduct on the right-hand side of Equation (3) is a yellowish-brown toxic gas, which is sparingly dissolved in water to produce the yellowish color of the RSN solution. Another byproduct of this reduction technique is NaOH, as shown on the right-hand side of Equation (3). Therefore, there are two contaminants present in the RSN solution: $C_{6}H_{8}O_{7}$ and NaOH. The process of RSN formation is depicted in Figure 5: (a) mixing and heating the precursor and reductor, (b) silver atom formation, and (c) silver nanoparticle production with ${NO}_{2}$ toxic gas byproduct.

The stock sample was prepared by adding 2 mL of 10 mM precursor and 2 mL of 10 mM stabilizer to a conical flask containing 36 mL of water. The concentration of silver was diluted by a factor of 1/20 to become 1079 ppm/20, which equals 54 ppm. This 54 ppm RSN solution was marked as the stock solution. The 30 ppm and 10 ppm RSN concentrations were diluted from this stock solution.

### 4.2. ESN Production

An electrolysis unit consisting of a 500 mL capacity of brown bottle, two identical silver plate electrodes, a black rubber bottle lid, a 24-volt DC power supply, 400 mL of distilled water, and two pieces of electrical cable were used to produce the ESN solution. The dimension of each silver plate was 16 cm in length, 4 cm in width, and 0.2 cm in thickness. The DC power supply was set at 24 V and 5 A.

Modifications were made to two parts of the electrolysis system. The first was replacing silver nitrate as the electrolyte with water. This was carried out because the source of the toxic gas NO_2_ was silver nitrate. However, by replacing silver nitrate as the electrolyte at the same time, the source of silver atoms was also lost. This led to the second modification, which was replacing the electrodes (which are usually a silver rod and a carbon rod) with two identical silver plates. Since two identical silver plates were used as electrodes, the electrical wiring connections could be made with any choice. The anode and cathode were interchangeable. Plates were chosen instead of rods to increase the surface area of the electrodes facing each other to increase the production of silver atoms. The replacement of silver nitrate solution as the electrolyte with water was chosen after carefully considering that the replacement of silver nitrate with silver chloride, silver bromide, or silver iodide also produces chlorine gas (Cl_2_), bromine gas (Br_2_), or iodine gas (I_2_), and all of them are toxic.

Ag^+^ ions released from the anode move to the cathode; some of them capture electrons in the solution to form silver atoms that remain in the solution to form ESNs, and some are neutralized by electrons on the cathode to coat the cathode surface. The equilibrium reaction of the production of ESN is given by:

(3) $$2{Ag}^{+}\left(aq\right)+2H_{2}O↔2Ag\left(aq\right)+H_{2}\left(g\right)+{2H}^{+}+O_{2}(g)$$

**Figure 6:**
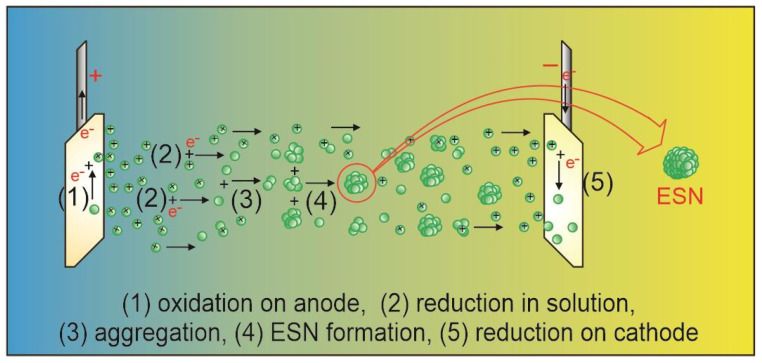
ESN production process: (1) oxidation on anode, (2) reduction in solution, (3) silver atom aggregation, (4) ESN formation, and (5) reduction on cathode. Green spheres with plus sign represent silver ions. Green single spheres represent silver atoms. Green spheres aggregates denote ESN.

The products on the right-hand side of Equation (3) are all gases except the silver atoms. H_2_ and O_2_ are friendly gases and return to nature. 2H^+^ turns into H_2_ gas as soon as it encounters electrons to form a covalent bond between the two H atoms. No contaminants or toxic gases are produced in this modified electrolysis. Figure 6 depicts ESN formation involving (1) oxidation producing silver ions in the anode, (2) reduction in solution and (5) in the cathode, resulting in silver atoms, (3) silver atom aggregation, and (4) ESN formation.

A stock of 400 mL of 31 ppm was produced within 2 h, as shown in Figure 2b. This stock sample was diluted to 30 ppm and 10 ppm using the simple equation $C_{1}V_{1}=C_{2}V_{2}$, where C_1_ is 31 ppm stock solution, V_1_ is the calculated stock volume in mL, C_2_ is the target concentration (30 ppm or 10 ppm), and V_2_ is the 100 mL target volume. The volume was V_1_ = (V_2_C_2_)/C_1_ = (100 mL × 30 ppm)/31 ppm = 96.77 mL. Therefore, 100 mL of the 30 ppm ESN sample was made by adding 96.77 mL of stock to 3.23 mL of water. A quantity of 100 mL of 10 ppm ESN was prepared in the same way.

The limitations of ESN production are basically its slow speed and the formation of a dark layer on the cathode. Slow production usually occurs after 30–40 min. The dark layer is formed immediately after the electrolysis process begins. The dark layer can be cleaned using alcohol or carbon cleaner. The slow production speed can be increased by installing multiple electrode pairs in parallel, using a larger electrode surface area, and spacing each electrode pair closer together.

### 4.3. Observation of Solution Color

The observation of solution colors was carried out by taking photographs of the 10 ppm and 30 ppm ESN solutions and 10 ppm and 30 ppm RSN solutions. All of them were taken in a single frame. Photographs were taken using a tablet Samsung S6 and converted into jpg, and finally, their resolution (dpi) was increased to 1200 dpi.

### 4.4. Detection of Silver in Solution

The presence of silver atoms in the solution was detected using UV–Visible spectrophotometry. The solution was scanned from 200 nm to 800 nm to find the absorption peak wavelength. The measurement is presented in the form of a graph of absorbance as a function of the wavelength of light. The presence of silver atoms or any other atoms can be identified by their peak wavelength. If 3 different types of atoms are present in the same solution, the graph shows 3 absorption peaks.

### 4.5. Particle Size Determination

The determination of particle size was conducted using the Laser Amplified Detection (LAD) method, a cutting-edge advancement of the Dynamic Light Scattering (DLS) technique [48]. Unlike UV–Visible spectrophotometry and other traditional light scattering systems, which require 4 mL of the sample, LAD requires only 5 microliters of the sample. Since the sample is very small, a precise sample preparation is needed. The presence of contaminant and aggregate in such a small size of the sample would destroy the measurement. In case this happens, the measurement must be repeated using a new sample taken from the same stock. The result of the measurement is presented in the form of a size distribution graph (Gaussian-like graph) with one peak and the value of the polydispersity index. The polydispersity index shows the homogeneity of the solution, and the peak of the Gaussian graph shows the mean diameter of the particles dispersed in the solution.

### 4.6. Antibacterial Activity Observation

The preparation of nutrient broth and nutrient agar was performed in the same way as in a previous publication [13]. The bacteria used in this research was *Propionibacterium acnes* ATCC 6919 strain NCTC 737 (VPI 0389). The antibacterial activity of ESNs (10 ppm and 30 ppm), ESNs (10 ppm and 30 ppm), and 5% chloramphenicol (positive control) was assessed in a Petri dish containing NA and *P. acnes*. The clear zone diameter was measured once in 3 hours at 3 different positions (horizontal, vertical, and diagonal) for a period of 72 h.

### 4.7. Statistical Analysis

All measurements were performed 3 times, and the data presented are the average of these three measurements, except for the DLS data, which were averaged from five measurements by the software. Statistical comparison of clear zone diameters between ESNs, RSNs, and chloramphenicol was performed individually using *t*-tests: two samples assuming unequal variances. *T*-test analyses were performed between 10 ppm ESN and 5% chloramphenicol, 30 ppm ESN and 5% chloramphenicol, 10 ppm RSN and 5% chloramphenicol, 30 ppm RSN and 5% chloramphenicol, 10 ppm ESN and 10 ppm RSN, 30 ppm ESN and 30 pm RSN, 30 ppm ESN and 10 ppm ESN, and 30 ppm RSN and 10 ppm RSN. The null hypothesis (Ho) of the comparison of 10 ppm ESN and 5% chloramphenicol was as follows: there is no significant difference in the clear zone diameter between 10 ppm ESN and 5% chloramphenicol. Therefore, the alternative hypothesis (Ha) was as follows: there is a significant difference in the clear zone diameter between 10 m ESN and 5% chloramphenicol. Both hypotheses were applied for all the comparisons above, and all *p*-values from the *t*-test analysis above are presented in Table 2.

## 5. Conclusions

In conclusion, the physical characteristics of ESNs are better than those of RSNs. The color of the ESN solution free of toxic gas is clear, like water, while the RSN solution is yellowish due to the dissolved yellow toxic gas NO_2_. The peak wavelength of ESN absorption is lower, which means there is no bathochromic auxochrome in the ESN solution, as can be seen from the analysis of the reaction products presented in Equation (3). The size of ESNs is much smaller, which means it can more easily penetrate the bacterial cell wall. The PDI of the ESN solution is also much lower, which indicates that ESN is much more homogeneous than RSN. Despite having many advantages, the electrolysis system has two problems that need to be overcome, namely the slow process and the appearance of a black layer on the cathode.

With pure (no surface contamination), small-sized, and homogeneous ESNs, further studies on the effect of coating on many physical and biological properties of silver nanoparticles can be successfully conducted. Without pure ESNs, coating studies cannot be successfully conducted due to the unintentional coating of byproducts such as citric acid during the production of silver nanoparticles. Cytotoxicity studies of silver nanoparticles should be conducted using pure ESNs to avoid the contribution of contaminant cytotoxicity.

The concentration dependence of ESN efficacy allows us to adjust the concentration of ESNs to obtain the desired efficacy to avoid the cytotoxicity of silver nanoparticles. The high efficacy of ESNs (the efficacy of 5% chloramphenicol is the same as that of 0.005% ESN) opens the opportunity to defeat more virulent bacteria, understand its efficacy against many different bacteria, and reveal the spectrum of ESNs against various bacteria.

The fact that *P. acnes* showed no resistance to pure ESNs (until the last observation at 72 h) opens the opportunity to use it against many other antibiotic-resistant bacteria. This may be the answer to the long-standing human effort to combat antibiotic-resistant bacteria.

## Figures and Tables

**Table 1:** Diameters of ESNs and RSNs.

Concentration (ppm)	ESN		RSN	
	Diameter (nm)	PDI	Diameter (nm)	PDI
10	40.3	0.0533	74	0.2848
30	39.9	0.0642	74.6	0.2948

**Table 2:** Two-tailed *p*-values of comparison chloramphenicol and both types of SN.

1	Chloramphenicol (%)	ESN (ppm)	RSN (ppm)	Two-Tailed *p*-Value
2	5	10	-	4.81 × 10^−18^
3	5	30	-	3.91 × 10^−8^
4	5	-	10	2.4 × 10^−9^
5	5	-	30	4.61 × 10^−16^
6	-	10	10	2.26 × 10^−13^
7	-	30	30	7.91 × 10^−20^
8	-	30:10	-	3.22 × 10^−14^
9	-	-	30:10	0.254671

## Data Availability

Data is contained within the article or Supplementary Material.

## Supplementary Materials

The following supporting information can be downloaded at: https://www.mdpi.com/article/10.3390/antibiotics14010086/s1, Data S1. containing DLS measurements of particle sizes and PDIs; Data S2. containing statistical analyses.
