# Supplementary material for: Antibacterial Efficacy Comparison of Electrolytic and Reductive Silver Nanoparticles Against Propionibacterium acnes
Source: Antibiotics (Basel). 2025 Jan 14;14(1):86. doi: 10.3390/antibiotics14010086 (PMC11759858; doi:10.3390/antibiotics14010086)
Supplement: Supplementary file 1 [file antibiotics-14-00086-s001.zip › antibiotics-3388238-supplementary.pdf]

Data S1

Supplementary material

Average particle size and PDI of ESN (10 ppm and 30 ppm) and RSN (10 ppm and 30 ppm)

Title: Antibacterial efficacy comparison of electrolytic and reductive silver nanoparticles against Propionibacterium acnes

Authors: Suparno Suparno[1,*], Rita Prasetyowati[1], Khafidh Nur Aziz[1], Anggarwati Rahma[1], Eka Sentia Ayu Lestari[1], Siti Nabiila[1], and Deby Grace[1]

Affiliation: [1] Universitas Negeri Yogyakarta, 1st Colombo St., Karangmalang, Depok, Yogyakarta 55281, Indonesia,

***Corresponding author. E-mail: suparno_mipa@uny.ac.id , Phone: +6281 2271 9098, fax: +62 274 548 203**

# - Particle Size Analysis -

C:\Microtrac\FLEX 11.1.0.6\Databases\Pak suparno.MDB

-Tabular Data -

Size (nm)	% Chan	% Pass	Size (nm)	% Chan	% Pass
6540	0.00	100.00	15.19	0.56	0.56
5500	0.00	100.00	12.77	0.00	0.00
4620	0.00	100.00	10.74	0.00	0.00
3890	0.00	100.00	9.03	0.00	0.00
3270	0.00	100.00	7.60	0.00	0.00
2750	0.00	100.00	6.39	0.00	0.00
2312	0.00	100.00	5.37	0.00	0.00
1944	0.00	100.00	4.52	0.00	0.00
1635	0.00	100.00	3.80	0.00	0.00
1375	0.00	100.00	3.19	0.00	0.00
1156	0.00	100.00	2.690	0.00	0.00
972.0	0.00	100.00	2.260	0.00	0.00
818.0	0.00	100.00	1.900	0.00	0.00
687.0	0.00	100.00	1.600	0.00	0.00
578.0	0.00	100.00	1.340	0.00	0.00
486.0	0.00	100.00	1.130	0.00	0.00
409.0	0.00	100.00	0.950	0.00	0.00
344.0	0.00	100.00			
289.0	0.00	100.00			
243.0	0.00	100.00			
204.4	0.00	100.00			
171.9	0.00	100.00			
144.5	0.00	100.00			
121.5	0.00	100.00			
102.2	0.00	100.00			
85.90	0.00	100.00			
72.30	1.03	100.00			
60.80	9.84	98.97			
51.10	27.72	89.13			
43.00	29.57	61.41			
36.10	16.44	31.84			
30.40	7.29	15.40			
25.55	3.69	8.11			
21.48	2.32	4.42			
18.06	1.54	2.10			

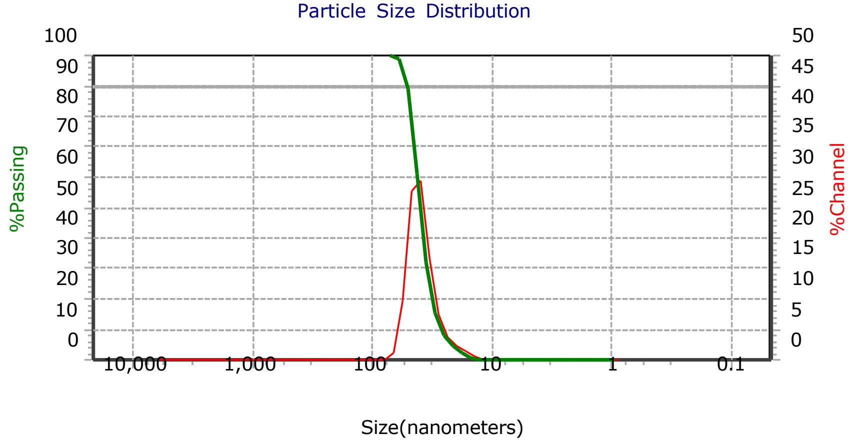

- Measurement Info -

Title	
SNP 10 ppm	
Identifiers	
SNP 10 ppm	
SNP 10 ppm	
Database Record	61
Run Number	AVG of 5
Date	12/6/2023
Time	12:13 PM
Acquired Date	12/6/2023
Acquired Time	12:13 PM
Serial Number	W3936
Calculated Data	
Above Residual	0
Below Residual	0
Loading Index	1.04E-2
Conc. Index : cc/m	0.00806 : 4.64E-5
RMS Residual	2.136%
Cell Temp (C)	24.37
Viscosity (cp)	0.9070
Reflected Pwr (uW)	3.00
User-Defined Calculations	
Name	Value
Recalculation Status	
DB-Meas : : Original :	

-SOP Info-

SNP 10 ppm	
Timing	
Set zero Time	60 (sec)
Run Time	30 (sec)
Number of Runs	5
Multi-Run Delay	0 (min)
Delay First Meas.	Disabled
Analysis	
SNP 10ppm	
Refractive Index	1.48
Transparency	Transp
Shape	Spherical
A1B1C3D2	
Refractive Index	1.38
Low Temperature	20.0
Low Temp. Visc.	1.002
High Temperature	30.0
High Temp. Visc.	0.797
Options:	
Analysis Type	Distribution
Filter:Resolution	Std:Norm
Sensitivity	Standard
Algorithm	2.0
Perspective	
Progression	Standard
Distribution	Intensity
Upper Edge (nm)	6540
Lower Edge (nm)	0.8
Residuals	Disabled

Summary	
Data	Value
MI (nm):	39.90
MN (nm):	29.58
MA (nm):	37.10
CS:	161.9
SD:	9.21
PDI:	0.0533
Mz:	40.03
si:	0.00957
Ski:	-0.07837
Kg:	1.090

Percentiles	
% Tile	Size (nm)
10.00	27.11
20.00	32.30
30.00	35.60
40.00	38.10
50.00	40.30
60.00	42.70
70.00	45.10
80.00	47.80
90.00	51.60
95.00	55.00

Dia (nm)	Vol %	Width
40.3	100	18.42

FLEX

11.1.0.6

- Notes -

-

12/8/2023 9:48 AM

# - Particle Size Analysis -

C:\Microtrac\FLEX 11.1.0.6\Databases\Pak suparno.MDB

-Tabular Data -

Size (nm)	% Chan	% Pass	Size (nm)	% Chan	% Pass
6540	0.00	100.00	15.19	0.00	0.00
5500	0.00	100.00	12.77	0.00	0.00
4620	0.00	100.00	10.74	0.00	0.00
3890	0.00	100.00	9.03	0.00	0.00
3270	0.00	100.00	7.60	0.00	0.00
2750	0.00	100.00	6.39	0.00	0.00
2312	0.00	100.00	5.37	0.00	0.00
1944	0.00	100.00	4.52	0.00	0.00
1635	0.00	100.00	3.80	0.00	0.00
1375	0.00	100.00	3.19	0.00	0.00
1156	0.00	100.00	2.690	0.00	0.00
972.0	0.00	100.00	2.260	0.00	0.00
818.0	0.00	100.00	1.900	0.00	0.00
687.0	0.00	100.00	1.600	0.00	0.00
578.0	0.00	100.00	1.340	0.00	0.00
486.0	0.00	100.00	1.130	0.00	0.00
409.0	0.00	100.00	0.950	0.00	0.00
344.0	0.00	100.00			
289.0	0.00	100.00			
243.0	1.58	100.00			
204.4	4.13	98.42			
171.9	7.23	94.29			
144.5	8.97	87.06			
121.5	9.38	78.09			
102.2	9.68	68.71			
85.90	10.49	59.03			
72.30	11.55	48.54			
60.80	11.86	36.99			
51.10	10.07	25.13			
43.00	6.95	15.06			
36.10	4.06	8.11			
30.40	2.24	4.05			
25.55	1.31	1.81			
21.48	0.50	0.50			
18.06	0.00	0.00			

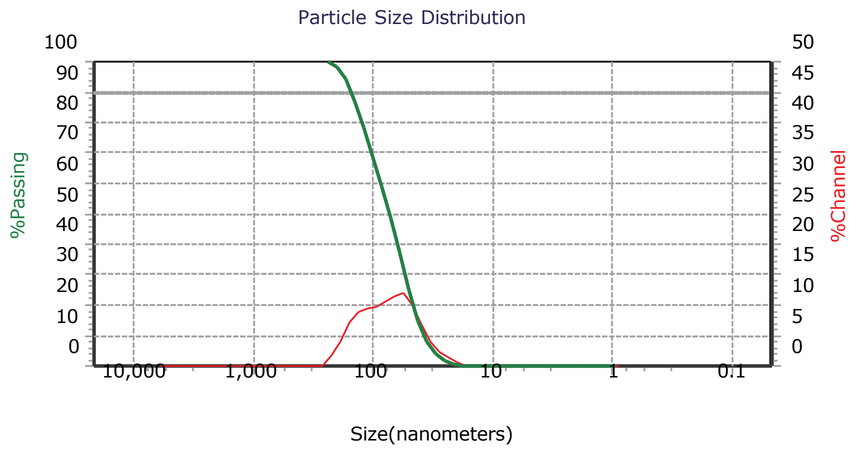

- Measurement Info -

Title	
RSN 10 ppm	
Identifiers	
RSN 10ppm	
RSN 10ppm	
Database Record	67
Run Number	Avg of 5
Date	16/11/2023
Time	09:20 PM
Acquired Date	16/11/202
Acquired Time	09:20 PM
Serial Number	W3936
Calculated Data	
Above Residual	0
Below Residual	0
Loading Index	5.31E-1
Conc. Index : cc/m	0.495 : 4.08E-4
RMS Residual	0.168%
Cell Temp (C)	24.31
Viscosity (cp)	0.9080
Reflected Pwr (uW)	3.00
User-Defined Calculations	
Name	Value
Recalculation Status	
DB-Meas : : Original :	

-SOP Info-

RSN 10 ppm	
Timing	
Set zero Time	60 (sec)
Run Time	30 (sec)
Number of Runs	5
Multi-Run Delay	0 (min)
Delay First Meas.	Disabled
Analysis	
RSN 10ppm	
Refractive Index	1.48
Transparency	Transp
Shape	Spherical
A1B1C3D2	
Refractive Index	1.38
Low Temperature	20.0
Low Temp. Visc.	1.002
High Temperature	30.0
High Temp. Visc.	0.797
Options:	
Analysis Type	Distribution
Filter:Resolution	Std:Norm
Sensitivity	Standard
Algorithm	2.0
Perspective	
Progression	Standard
Distribution	Intensity
Upper Edge (nm)	6540
Lower Edge (nm)	0.8
Residuals	Disabled

Summary	
Data	Value
MI (nm):	86.30
MN (nm):	40.60
MA (nm):	65.60
CS:	91.45
SD:	46.10
PDI:	0.2848
Mz:	84.59
si:	0.0449
Ski:	0.380
Kg:	0.926

Percentiles	
% Tile	Size (nm)
10.00	38.20
20.00	47.10
30.00	54.90
40.00	63.50
50.00	74.00
60.00	87.40
70.00	104.7
80.00	125.9
90.00	154.1
95.00	176.0

Dia (nm)	Vol %	Width
74	100	92.2

FLEX

11.1.0.6

- Notes -

-

16/11/2023 9:45 AM

Microtrac — Total Solutions in Particle Characterization

# - Particle Size Analysis -

C:\Microtrac\FLEX 11.1.0.6\Databases\Pak suparno.MDB

-Tabular Data -

Size (nm)	% Chan	% Pass	Size (nm)	% Chan	% Pass
6540	0.00	100.00	15.19	0.00	0.00
5500	0.00	100.00	12.77	0.00	0.00
4620	0.00	100.00	10.74	0.00	0.00
3890	0.00	100.00	9.03	0.00	0.00
3270	0.00	100.00	7.60	0.00	0.00
2750	0.00	100.00	6.39	0.00	0.00
2312	0.00	100.00	5.37	0.00	0.00
1944	0.00	100.00	4.52	0.00	0.00
1635	0.00	100.00	3.80	0.00	0.00
1375	0.00	100.00	3.19	0.00	0.00
1156	0.00	100.00	2.690	0.00	0.00
972.0	0.00	100.00	2.260	0.00	0.00
818.0	0.00	100.00	1.900	0.00	0.00
687.0	0.00	100.00	1.600	0.00	0.00
578.0	0.00	100.00	1.340	0.00	0.00
486.0	0.00	100.00	1.130	0.00	0.00
409.0	0.00	100.00	0.950	0.00	0.00
344.0	0.00	100.00			
289.0	0.00	100.00			
243.0	1.58	100.00			
204.4	4.13	98.42			
171.9	7.23	94.29			
144.5	8.97	87.06			
121.5	9.38	78.09			
102.2	9.68	68.71			
85.90	10.49	59.03			
72.30	11.55	48.54			
60.80	11.86	36.99			
51.10	10.07	25.13			
43.00	6.95	15.06			
36.10	4.06	8.11			
30.40	2.24	4.05			
25.55	1.31	1.81			
21.48	0.50	0.50			
18.06	0.00	0.00			

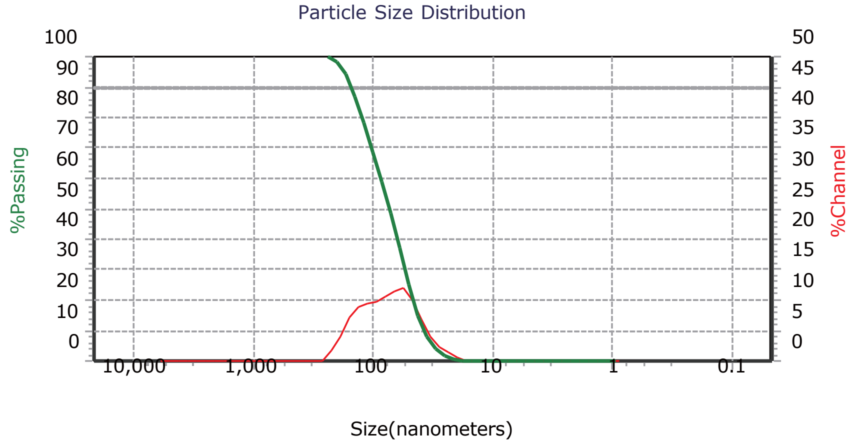

- Measurement Info -

Title	
RSN 10 ppm	
Identifiers	
RSN 10 ppm	
RSN 10 ppm	
Database Record	67
Run Number	Avg of 5
Date	16/11/2023
Time	09:20 PM
Acquired Date	16/11/202
Acquired Time	09:20 PM
Serial Number	W3936
Calculated Data	
Above Residual	0
Below Residual	0
Loading Index	5.31E-1
Conc. Index : cc/m	0.495 : 4.08E-4
RMS Residual	0.168%
Cell Temp (C)	24.31
Viscosity(cp)	0.9080
Reflected Pwr (uW)	3.00
User-Defined Calculations	
Name	Value
Recalculation Status	
DB-Meas : : Original :	

-SOP Info-

RSN 10 ppm	
Timing	
Set zero Time	60 (sec)
Run Time	30 (sec)
Number of Runs	5
Multi-Run Delay	0 (min)
Delay First Meas.	Disabled
Analysis	
RSN 10ppm	
Refractive Index	1.48
Transparency	Transp
Shape	Spherical
A1B1C3D2	
Refractive Index	1.38
Low Temperature	20.0
Low Temp. Visc.	1.002
High Temperature	30.0
High Temp. Visc.	0.797
Options:	
Analysis Type	Distribution
Filter:Resolution	Std:Norm
Sensitivity	Standard
Algorithm	2.0
Perspective	
Progression	Standard
Distribution	Intensity
Upper Edge (nm)	6540
Lower Edge (nm)	0.8
Residuals	Disabled

Summary	
Data	Value
MI (nm):	86.30
MN (nm):	40.60
MA (nm):	65.60
CS:	91.45
SD:	46.10
PDI:	0.2848
Mz:	84.59
si:	0.0449
Ski:	0.380
Kg:	0.926

Percentiles	
% Tile	Size (nm)
10.00	38.20
20.00	47.10
30.00	54.90
40.00	63.50
50.00	74.00
60.00	87.40
70.00	104.7
80.00	125.9
90.00	154.1
95.00	176.0

Dia (nm)	Vol %	Width
74	100	92.2

FLEX

11.1.0.6

- Notes -

-

16/11/2023 9:45 AM

![Microtrac logo] Total Solutions in Particle Characterization

# - Particle Size Analysis -

C:\Microtrac\FLEX 11.1.0.6\Databases\Pak suparno.MDB

-Tabular Data -

Size (nm)	% Chan	% Pass	Size (nm)	% Chan	% Pass
6540	0.00	100.00	15.19	0.00	0.00
5500	0.00	100.00	12.77	0.00	0.00
4620	0.00	100.00	10.74	0.00	0.00
3890	0.00	100.00	9.03	0.00	0.00
3270	0.00	100.00	7.60	0.00	0.00
2750	0.00	100.00	6.39	0.00	0.00
2312	0.00	100.00	5.37	0.00	0.00
1944	0.00	100.00	4.52	0.00	0.00
1635	0.00	100.00	3.80	0.00	0.00
1375	0.00	100.00	3.19	0.00	0.00
1156	0.00	100.00	2.690	0.00	0.00
972.0	0.00	100.00	2.260	0.00	0.00
818.0	0.00	100.00	1.900	0.00	0.00
687.0	0.09	100.00	1.600	0.00	0.00
578.0	0.35	99.91	1.340	0.00	0.00
486.0	0.67	99.56	1.130	0.00	0.00
409.0	1.11	98.89	0.950	0.00	0.00
344.0	1.33	97.78			
289.0	1.57	96.45			
243.0	2.12	94.88			
204.4	3.35	92.76			
171.9	4.64	89.41			
144.5	5.99	84.77			
121.5	8.06	78.78			
102.2	10.63	70.72			
85.90	12.32	60.09			
72.30	12.56	47.77			
60.80	11.54	35.21			
51.10	9.37	23.67			
43.00	6.59	14.30			
36.10	4.07	7.71			
30.40	2.28	3.64			
25.55	1.18	1.36			
21.48	0.18	0.18			
18.06	0.00	0.00			

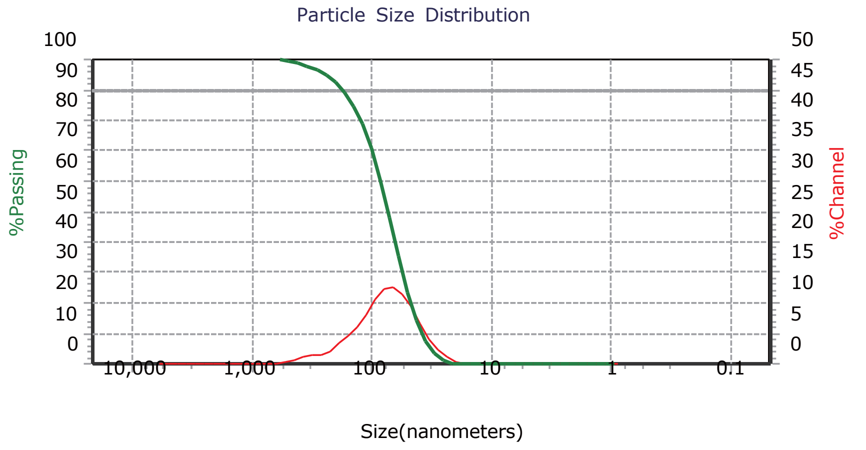

- Measurement Info -

Title	
RSN 30 ppm	
Identifiers	
RSN 30 ppm	
RSN 30 ppm	
Database Record	66
Run Number	Avg Of 5
Date	16/11/2023
Time	09:13 AM
Acquired Date	16/11/2023
Acquired Time	09:13 AM
Serial Number	W3936
Calculated Data	
Above Residual	0
Below Residual	0
Loading Index	3.14E-1
Conc. Index : cc/m	0.304 : 3.26E-4
RMS Residual	0.125%
Cell Temp (C)	25.05
Viscosity (cp)	0.8930
Reflected Pwr (uW)	3.00
User-Defined Calculations	
Name	Value
Recalculation Status	
DB-Meas : : Original :	

-SOP Info-

RSN 30 ppm	
Timing	
Set zero Time	60 (sec)
Run Time	30 (sec)
Number of Runs	5
Multi-Run Delay	0 (min)
Delay First Meas.	Disabled
Analysis	
RSN 30ppm	
Refractive Index	1.48
Transparency	Transp
Shape	Spherical
A1B1C3D2	
Refractive Index	1.38
Low Temperature	20.0
Low Temp. Visc.	1.002
High Temperature	30.0
High Temp. Visc.	0.797
Options:	
Analysis Type	Distribution
Filter:Resolution	Std:Norm
Sensitivity	Standard
Algorithm	2.0
Perspective	
Progression	Standard
Distribution	Intensity
Upper Edge (nm)	6540
Lower Edge (nm)	0.8
Residuals	Disabled

Summary	
Data	Value
MI (nm):	96.80
MN (nm):	42.10
MA (nm):	67.30
CS:	89.15
SD:	48.20
PDI:	0.2948
Mz:	86.71
si:	0.0565
Ski:	0.492
Kg:	1.482

Percentiles	
% Tile	Size (nm)
10.00	38.70
20.00	48.00
30.00	56.40
40.00	65.00
50.00	74.60
60.00	85.80
70.00	100.9
80.00	125.5
90.00	176.6
95.00	246.0

Dia (nm)	Vol %	Width
74.6	100	96.5

FLEX

11.1.0.6

- Notes -

-

16/11/2023 9:47 AM

Data S2

Supplementary material

Statistical analysis complete comparison between ESN and RSN (10 ppm and 30 ppm); between ESN and chloramphenicol 5%; between RSN and chloramphenicol 5%

Title: Antibacterial efficacy comparison of electrolytic and reductive silver nanoparticles against Propionibacterium acnes

Authors: Suparno Suparno[1,*], Rita Prasetyowati[1], Khafidh Nur Aziz[1], Anggarwati Rahma[1], Eka Sentia Ayu Lestari[1], Siti Nabiila[1], and Deby Grace[1]

Affiliation: [1] Universitas Negeri Yogyakarta, 1st Colombo St., Karangmalang, Depok, Yogyakarta 55281, Indonesia,

*__Corresponding author. E-mail: suparno_mipa@uny.ac.id , Phone: +6281 2271 9098, fax: +62 274 548 203__

1. t-Test: Two-sample Assuming Unequal Variances

Comparison (a) between chloramphenicol and 10 ppm ESN;

(b) between chloramphenicol and 10 ppm RSN;

(c) between 10 ppm ESN and 10 ppm RSN.

Title: Antibacterial activity of silver nanoparticles produced by electrolysis and reduction method against Propionibacterium acnes

Authors: Suparno, Suparno et al.

Time	RSN 10	ESN 10	Chloram.
3			
6			
9			Chlo.
12	CS 10	SN 10	8.433333
15	7.166667	8	8.333333
18	7.233333	8.033333	8.166667
21	7.133333	8.133333	8.333333
24	7.066667	8.033333	8.266667
27	7.133333	8.066667	8.166667
30	7.166667	7.933333	8.233333
33	7.266667	7.933333	8.266667
36	7.033333	7.733333	8.133333
39	7.033333	7.766667	8.133333
42	6.966667	7.733333	0
45	7.266667	7.666667	0
48	7.133333	7.733333	0
51	7.166667	7.633333	0
54	7.133333	7.533333	0
57	7.433333	7.533333	0
60	7.266667	7.566667	0
63	7.433333	7.833333	0
66	7.233333	7.666667	0
69		7.666667	0
72		7.633333	0
AVG.	7.181481	7.807407	8.246667
SDev.	0.121829	0.185255	0.094516

(a) Comparison between Chloramphenicol and 10 ppm ESN

t-Test: Two-Sample Assuming Unequal V riances

	Chloram.	*ESN 10*
Mean	8.246667	7.791667
Variance	0.009926	0.036126
Observations	10	20
Hypothesized Me	0	
df	28	
t Stat	8.600413	
P(T<=t) one-tail	1.2E-09	
t Critical one-tail	1.701131	
P(T<=t) two-tail	2.4E-09	
t Critical two-tail	2.048407	

P value = 2.4 x 10(-9) < 0.05

Ho is rejected so that Chloramphenicol produced significantly larger clear zone diameter than 10 ppm RSN

(b) Comparison between Chloramphenicol and 10 ppm RSN

t-Test: Two-Sample Assuming Unequal V riances

	Chloram.	*RSN 10*
Mean	8.246667	7.181481
Variance	0.009926	0.015715
Observations	10	18
Hypothesized Me	0	
df	23	
t Stat	24.66086	
P(T<=t) one-tail	2.4E-18	
t Critical one-tail	1.713872	
P(T<=t) two-tail	4.81E-18	
t Critical two-tail	2.068658	

P-value = 4.81 x 10(-18) < 0.05

Ho is rejected, so chloramphenicol produced significantly larger clear zone diameter than 10 ppm ESN

(c) Comparison between 10 ppm ESN and 10 ppm RSN

t-Test: Two-Sample Assuming Unequal Variances

	ESN 10	*RSN 10*
Mean	7.791667	7.181481481
Variance	0.036126	0.015715323
Observatio	20	18
Hypothesiz	0	
df	33	
t Stat	11.78816	
P(T<=t) on	1.13E-13	
t Critical o	1.69236	
P(T<=t) tw	2.26E-13	
t Critical tw	2.034515	

P-value = 2.26 x 10(-13) < 0.05

Ho is rejected, so 10 ppm ESN produced

significantly larger clear zone diameter than 10 ppm RSN

2. t-Test analysis comparison (a) between 5% chloramphenicol and 30 ppm ESN
(b) between 5% chloramphenicol and 30 ppm RSN
(c) between 30 ppm ESN and 30 ppm RSN

Title: Antibacterial activity of silver nanoparticles produced by electrolysis and reduction method against Propionibacterium acnes

Authors: Suparno, Suparno et al.

Time	RSN30	ESN30	Chloram.
3			
6			
9			Chlo.
12	CS30	SN30	9.133333
15	7.13333333	8.533	9.566667
18	7.63333333	8.667	9.333333
21	7.46666667	8.567	9.133333
24	7.63333333	8.533	9.233333
27	7.13333333	8.233	9.133333
30	7.06666667	8.533	9.166667
33	7.13333333	8.267	9.033333
36	7.16666667	8.333	9.033333
39	6.93333333	8.367	
42	7.06666667	8.467	
45	7.13333333	8.433	
48	7.23333333	8.333	
51	7.33333333	8.467	
54	7.16666667	8.267	
57	7.43333333	8.267	
60	7.33333333	8.533	
63	7.23333333	8.533	
66	7.16666667	8.433	
69		8.567	
Sdev.	0.18724777	0.121324	0.157484

2 (a) t-Test: Two-Sample Assuming Unequal Variances between 5% Chloramphenicol and 30 ppm ESN

	Chloram.	*ESN30*
Mean	9.196296	8.435
Variance	0.027901	0.015494
Observations	9	20
Hypothesized M	0	
df	12	
t Stat	12.23	
P(T<=t) one-tail	1.96E-08	
t Critical one-tail	1.782288	
P(T<=t) two-tail	3.91E-08	
t Critical two-tail	2.178813	

P value= 3.91 x 10(-8) < 0.05
Ho is rejected so, Chloramphenicol produced significantly higher clear zone diameter than 30 ppm ESN

2 (b) t-Test: Two-Sample Assuming Unequal Variances between 5% Chloramphenicol and 30 ppm RSN

	Chloram.	*RSN30*
Mean	9.196296	7.244444
Observations	9	18
Hypothesized M	0	
df	18	
t Stat	27.1652	
P(T<=t) one-tail	2.31E-16	
t Critical one-tail	1.734064	
P(T<=t) two-tail	4.61E-16	
t Critical two-tail	2.100922	

P-value= 4.61 x 10 (-16) < 0.05
Ho is rejected, so Chloramphenicol produced significantly higher clear zone diameter than 30 ppm RSN

2 (c) t-Test: Two-Sample Assuming Unequal Variances between 30 ppm ESN and 30 ppm RSN

	ESN30	*RSN30*
Mean	8.435	7.244444
Variance	0.015494	0.037124
Observations	20	18
Hypothesized	0	
df	29	
t Stat	22.35155	
P(T<=t) one-t	3.95E-20	
t Critical one-	1.699127	
P(T<=t) two-t	7.91E-20	
t Critical two-	2.04523	

P-value= 7.91 x 10 (-20) < 0.05
Ho is rejected, so 30 ppm SN produced
significantly higher clear zone diameter than
30 ppm RSN
